# Richardson Syndrome Variant of Progressive Supranuclear Palsy: A Case Report

**DOI:** 10.7759/cureus.98307

**Published:** 2025-12-02

**Authors:** Ibrahim Korucu, Tuba Karakoyun Alpay

**Affiliations:** 1 Department of Neurology, Edirne Sultan 1. Murat State Hospital, Edirne, TUR; 2 Department of Neurology, Cerkezkoy State Hospital, Tekirdag, TUR

**Keywords:** atypical parkinsonism, movement disorder therapy, neurodegenerative diseases, progressive supranuclear palsy-richardson syndrome (psp-rs), tauopathy

## Abstract

Progressive supranuclear palsy (PSP) is a chronic neurodegenerative disease, an atypical parkinsonism with four-repeat tau neuropathology. PSP-Richardson syndrome (PSP-RS) is the most prevalent clinical variant of PSP, originally described as the prototypical form of the disease. It is defined by vertical supranuclear gaze palsy (most prominently affecting downward gaze), early postural instability with frequent falls, and a subcortical-frontal pattern of cognitive impairment. The diagnosis of PSP is mainly based on clinical data and is only definitively confirmed at autopsy. Neuroimaging plays an important role in early diagnosis.

Our report describes the case of a 72-year-old female patient who had experienced subtle symptoms for approximately two years prior to presentation. Initially, she exhibited slow and monotonous speech, mild decline in fine motor skills, intermittent episodes of impaired concentration, and mild forgetfulness. At presentation to the outpatient clinic, she reported falls, imbalance, bradykinesia, cognitive decline, limitation of vertical gaze, and diplopia. The patient's systemic examination was normal. Neurological examination showed vertical gaze palsy, postural instability, bradykinesia, and a shuffling gait. Bilateral upper extremity associative movements were slow. The Mini-Mental State Examination Score was 19. Axial T2-weighted magnetic resonance imaging (MRI) showed midbrain atrophy, as well as the hummingbird sign and morning glory sign, characteristic of PSP. Axial T1 MRI showed cerebral atrophy and dilatation of the bilateral lateral and third ventricles. Initially, levodopa/benserazide was started at a dose of 500 mg/day and increased up to 1,000 mg/day. Amantadine at a dose of 200 mg/day and levodopa/carbidopa sustained release at a dose of 200/50 mg/day were added to the treatment regimen, but no effective clinical response was observed. Balance and walking exercises were provided by the physical therapy and rehabilitation department.

This case highlights the subtle prodromal features and progressive course of PSP, emphasizing the importance of early recognition. It also contributes to the literature by illustrating the clinical progression and therapeutic challenges in managing PSP, underscoring the value of a multidisciplinary approach to optimize patient outcomes.

## Introduction

Progressive supranuclear palsy (PSP) is a chronic neurodegenerative disease, an atypical parkinsonism with four-repeat tau neuropathology. Since the seminal description of the disorder by Steele, Richardson, and Olszewski in 1964, conceptual and pathological insights into PSP have markedly evolved. PSP-Richardson syndrome (PSP-RS) is the most prevalent clinical variant of PSP, originally described as the prototypical form of the disease. It is defined by vertical supranuclear gaze palsy (most prominently affecting downward gaze), early postural instability with frequent falls, and a subcortical-frontal pattern of cognitive impairment. PSP-RS is classified as a rare disease with an incidence of approximately five to seven cases per 100,000 people [1]. A recent study in the United Kingdom found that the incidence peaked at 18 cases per 100,000, occurring in the age group of 70 to 74 years [2]. A Japanese study that included other PSP phenotypes, along with PSP-RS, found an incidence of 18 cases per 100,000 across all ages [3]. Yoshida et al. found a higher-than-expected prevalence of early stage PSP in a study of forensic cases. When applied to larger population-based cohorts, these findings suggest that the prevalence of PSP may be higher than previously estimated [4]. The diagnosis of PSP is mainly based on clinical data and is only definitively confirmed at autopsy. Neuroimaging plays an important role in early diagnosis [5].

## Case presentation

**Figure 1:**
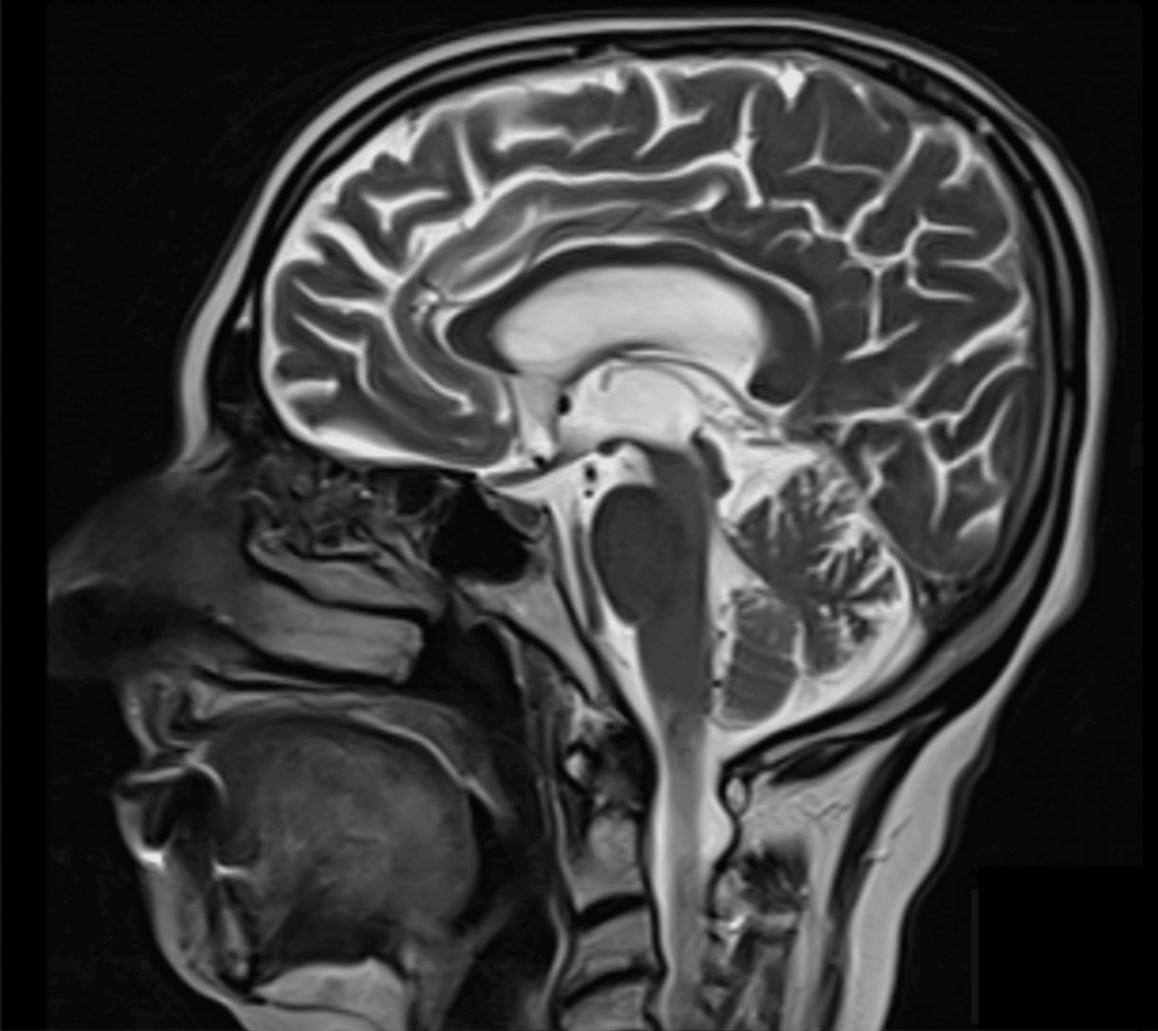
The sagittal T2-weighted MRI showing selective and pronounced atrophy of the midbrain, with mesencephalic predominance and preservation of the pons, characteristic of the “hummingbird sign” in PSP. The midbrain-pons ratio is 0.43 (which, when less than 0.52, demonstrates 100% specificity for PSP). The midbrain-pons area ratio is 0.14 (a value less than 0.16 is suggestive of PSP). PSP, progressive supranuclear palsy

**Figure 2:**
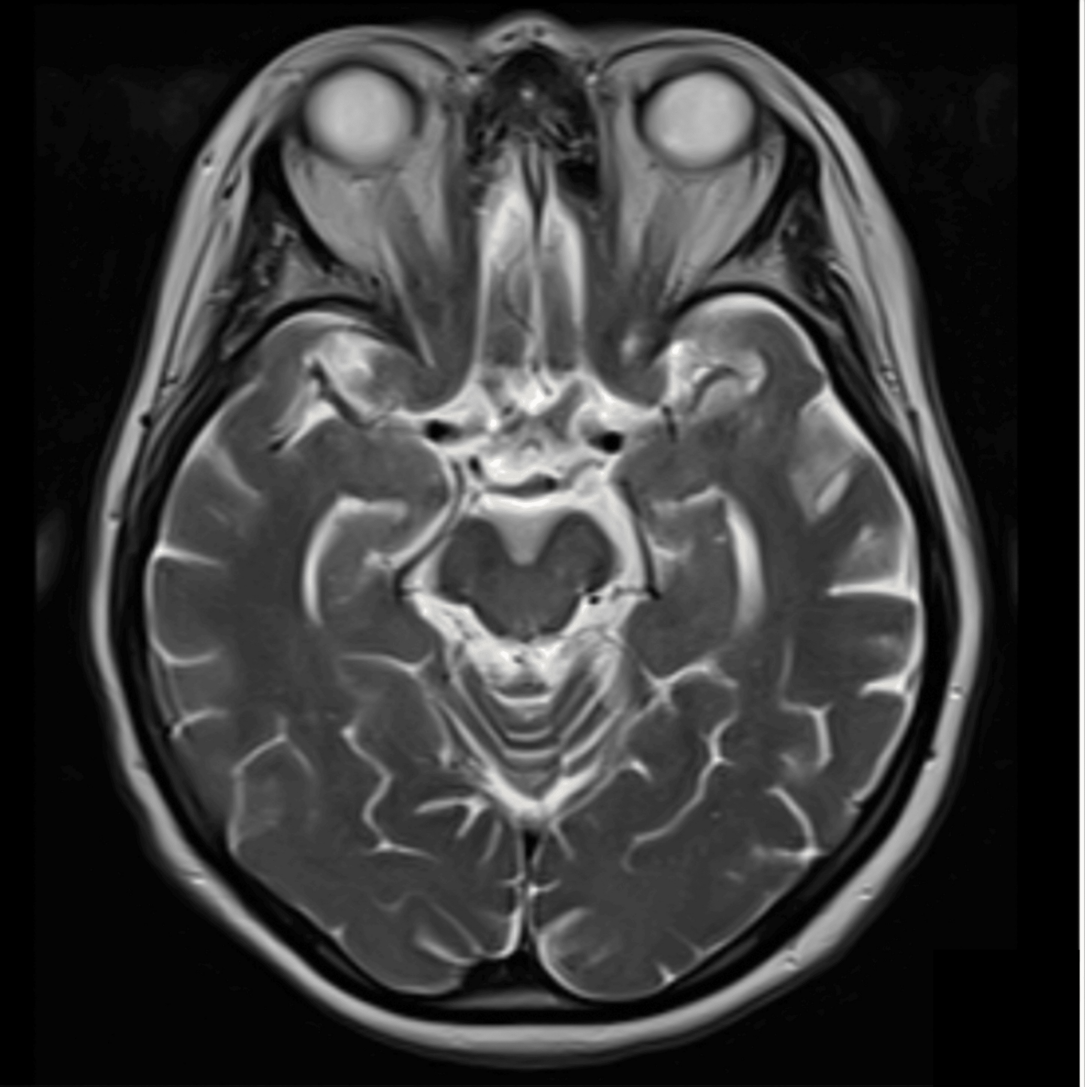
Axial T2-weighted MRI showing atrophy of the midbrain tegmentum, manifesting as a reduced anteroposterior midbrain diameter and thinning of the cerebral peduncles. These changes produce the distinctive “morning glory” and “Mickey Mouse” signs, which are hallmark imaging features of PSP. PSP, progressive supranuclear palsy

**Figure 3:**
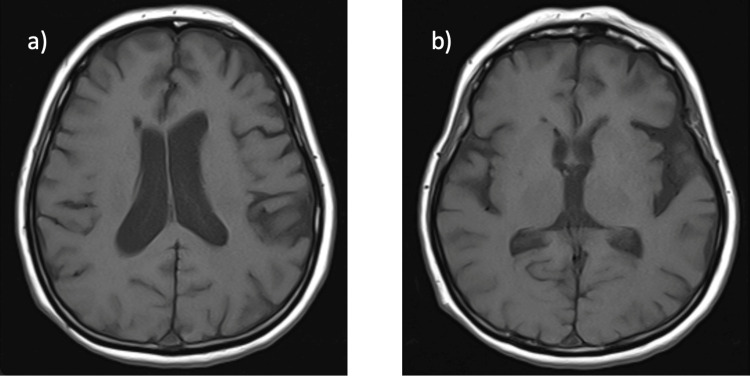
The axial T1 MRI showing cerebral atrophy, bilateral lateral ventricle dilatation (a), and third ventricle dilatation (b).

Our report describes the case of a 72-year-old female patient who had experienced subtle symptoms for approximately two years prior to presentation. Initially, she exhibited slow and monotonous speech, mild decline in fine motor skills, particularly difficulty with buttoning clothing, intermittent episodes of impaired concentration, and mild forgetfulness. These early manifestations were subtle and fluctuating, gradually progressing to more overt motor and cognitive deficits. At presentation to the outpatient clinic, she reported falls, imbalance, bradykinesia, cognitive decline, limitation of vertical gaze, and diplopia. Her medical history included only hypertension. The patient's systemic examination was normal. Neurological examination showed vertical gaze palsy, postural instability, bradykinesia, and a shuffling gait. Bilateral upper extremity associative movements were slow. The Mini-Mental State Examination Score was 19. Axial T2-weighted magnetic resonance imaging (MRI) showed midbrain atrophy, as well as the hummingbird sign and morning glory sign, characteristic of PSP. The midbrain-pons ratio was 0.43. On the midsagittal T1-weighted MRI slice, the maximum diameter perpendicular to the major axis was measured for both the pons and the midbrain; the pons measurement excluded the tegmentum, while the midbrain measurement excluded the collicular plate [6]. A ratio below 0.52 has been reported to demonstrate a very high specificity for PSP [7]. The midbrain-to-pons area ratio was 0.14. For this measurement, the areas of the midbrain (excluding the collicular plate) and pons (excluding the tegmentum) were manually outlined on midsagittal T1-weighted MRI, and their respective surface areas were calculated; the ratio was obtained by dividing the midbrain area by the pons area [7]. A value less than 0.16 is suggestive of PSP (Figures 1, 2). Axial T1 MRI showed cerebral atrophy and dilatation of the bilateral lateral and third ventricles (Figure 3). Levodopa/benserazide was initiated at 500 mg/day and gradually increased to 1,000 mg/day. Amantadine 200 mg/day and levodopa/carbidopa sustained release 200/50 mg/day were added, but no effective clinical response was observed. Balance and walking exercises were provided by the physical therapy and rehabilitation department. During the one-year follow-up period, the patient exhibited a progressive clinical decline. The frequency of falls increased significantly, resulting in traumatic injuries including a clavicular fracture and a radial fracture. Cognitive deterioration also accelerated, with further worsening of executive dysfunction and memory impairment, consistent with the natural progression of PSP.

## Discussion

In PSP, early diagnosis and a multidisciplinary approach play an important role in the patient's treatment and rehabilitation process. At present, there is no available therapy that alters the course of PSP. Existing treatment approaches are primarily symptomatic, aiming to alleviate clinical manifestations and enhance the patient’s ability to perform daily activities. In general, the pharmacological approach to PSP is based on experience and uncontrolled case series, rather than on well-conducted controlled clinical trials. Drugs that have shown benefit in neurodegenerative diseases, particularly Parkinson disease (PD), are used [2, 8-9].

Current pharmacological treatments for PSP are symptomatic and mild to moderately effective. Levodopa is administered to manage bradykinesia and rigidity. In a retrospective study of pathologically confirmed PSP patients, 32% exhibited over 30% improvement on the Unified Parkinson Disease Rating Scale (UPDRS), whereas 4% experienced levodopa-induced dyskinesia [10]. Similar response rates have been observed across multiple studies [11,12]. However, clinical responses to levodopa are generally lower than those to PD, and higher doses of levodopa are required to achieve the same response. Clinical response may worsen over time. Titration to 1,000 mg/day and maintenance of this dose for at least one month is recommended [12]. A marked and sustained clinical response to levodopa is regarded as an exclusion criterion for PSP, indicating that a diagnosis of PD is more probable [13,14]. Dopamine agonists have been evaluated in PSP; however, they are generally less effective than levodopa and are associated with a higher frequency of adverse effects. Multiple investigations have demonstrated that amantadine administration in PSP is associated with amelioration of bradykinesia, rigidity, and dystonia. However, side effects of varying severity have been reported, including pedal and pretibial edema, livedo reticularis, hallucinations, and worsening cognitive impairment. If dose-limiting side effects occur, it is recommended to taper the drug by 100 mg per week, as delirium withdrawal syndrome has been described with abrupt discontinuation of amantadine [15]. In line with previously reported literature, high-dose levodopa and amantadine therapy was initiated in the patient; however, as expected, no significant clinical response was observed.

Some clinicians suggest that bromocriptine may provide a modest benefit in certain PSP patients; however, its efficacy is generally minimal and transient. Tricyclic antidepressants have also been employed, and other agents such as trazodone have been tested, though with limited success [2].

In patients with PSP, the leading causes of death are infections and pulmonary complications, such as pneumonia, often secondary to prolonged immobility. The predominant source of morbidity is typically postural instability resulting in immobility, although dementia, visual disturbances, and dysphagia also contribute significantly. Approximately 50% of patients require walking assistance within three years of symptom onset. On average, the progression from initial symptom manifestation to the need for a cane or walker is about 3.1 years, while confinement to a chair or bed occurs after roughly 8.2 years [2,14].

OnabotulinumtoxinA has demonstrated efficacy in managing rigidity, particularly nuchal rigidity, and various forms of dystonia, including bruxism, blepharospasm, and focal limb dystonia. It may also offer therapeutic benefit in reducing sialorrhea [16].

Patients are advised to follow a balanced diet. When oral intake is no longer viable due to elevated risks of dysphagia and aspiration, gastrostomy should be regarded as an appropriate method for ensuring adequate nutrition [17].

A randomized, placebo-controlled, double-blind, phase 2 study found that supplemental coenzyme Q10 at a dose of 5 mg/kg per day for six weeks resulted in favorable changes in brain energy metabolites and modest but significant improvements in some measures of motor and neuropsychological dysfunction [18].

Gait impairment and recurrent falls represent two of the primary contributors to disability in patients with PSP. Following gait assessment, interventions such as physiotherapy, occupational therapy aimed at optimizing mobility strategies, structured exercise programs, and the use of suitable mobility aids can help decrease fall frequency and associated complications. In our patient, the frequency of falls increased significantly, leading to traumatic injuries, including a clavicular fracture and a radial fracture. These events illustrate the vulnerability of PSP patients to a “fracture cascade,” in which recurrent falls precipitate sequential skeletal injuries. Such fractures not only contribute to morbidity and functional decline but also underscore the importance of proactive fall prevention strategies and bone health management in this population [19].

Our case initially exhibited mild prodromal features of PSP (slow and monotonous speech, mild regression in fine motor skills, intermittent attention deficits, and mild forgetfulness). These early symptoms, consistent with prodromal symptoms reported in the literature, gradually evolved into more pronounced motor and cognitive deficits, highlighting the insidious onset and progressive nature of the disease [20,21].

## Conclusions

PSP is frequently misdiagnosed due to its heterogeneous presentation. Accurate diagnosis necessitates heightened clinical awareness, emphasizing the importance of a thorough understanding of the disease for timely recognition. This case highlights the insidious onset and progressive nature of PSP, as well as the challenges associated with its clinical management. Early recognition of subtle prodromal symptoms, vigilant monitoring for motor and cognitive decline, and proactive interventions for complications such as falls and fractures are essential. Furthermore, a multidisciplinary approach - integrating neurology, physiotherapy, occupational therapy, nutrition, and, when appropriate, pharmacological management - plays a critical role in optimizing patient outcomes and improving both patient and caregiver quality of life.
